# Supplementary material for: MLL5 suppresses antiviral innate immune response by facilitating STUB1-mediated RIG-I degradation
Source: Nat Commun. 2018 Mar 28;9:1243. doi: 10.1038/s41467-018-03563-8 (PMC5871759; doi:10.1038/s41467-018-03563-8)
Supplement: Supplementary file 1 — Supplementary Information(PDF 1587 kb) [file 41467_2018_3563_MOESM1_ESM.pdf]

MLL5 suppresses antiviral innate immune response by facilitating STUB1-mediated RIG-I degradation

Zhou et al.

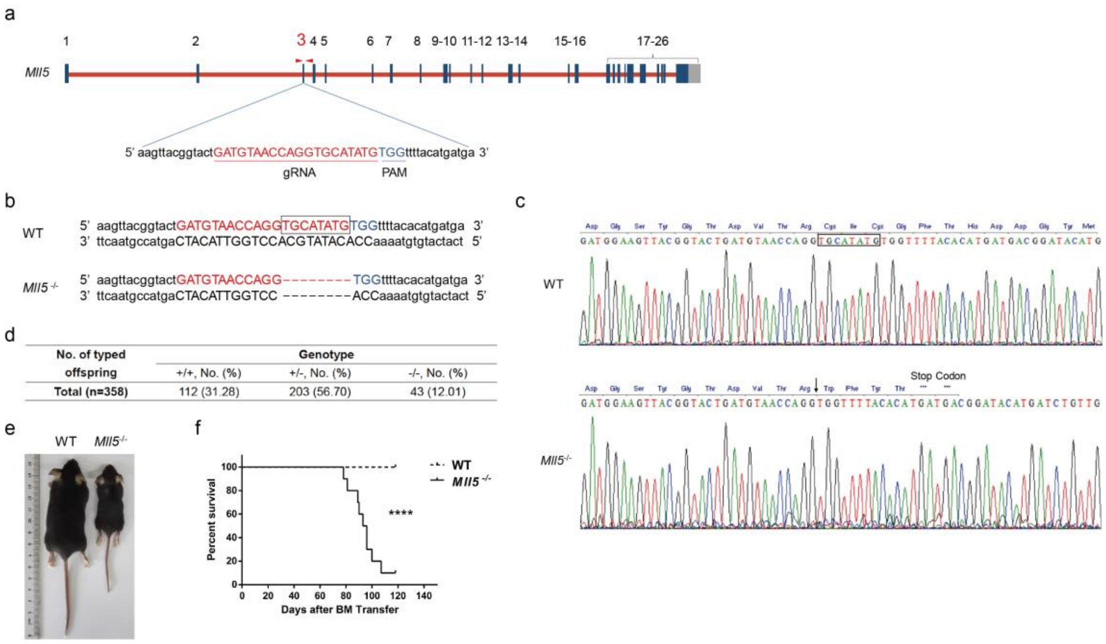

Supplementary Figure 1. Generation of *Mll5*-deficient mice with CRISPR-Cas9 approach. **(a)** Graphic representation of strategy used to generate *Mll5*$^{-/-}$ mice. gRNA (red) and PAM (blue) sequences locus in exon 3 of *Mll5* gene. **(b)** DNA sequence of WT (upper) and *Mll5*$^{-/-}$ mice (bottom). The region of the target sequence is shown in red. The black box indicates the nucleotides lost in *Mll5*$^{-/-}$ mice. **(c)** Sequence analysis of the the Reverse Transcription-PCR band in MEFs from WT (upper) and *Mll5*$^{-/-}$ mice (bottom). The nucleotide sequence confirms the reading frame shift in exon 3 in *Mll5*$^{-/-}$ mice; a premature stop codon (TGA) in exon 3 is induced. **(d)** Number and frequency of WT, *Mll5*$^{+/-}$ and *Mll5*$^{-/-}$ pups by *Mll5*$^{+/-}$ intercrosses at the time of weaning. **(e)** Postnatal growth retardation in *Mll5*-deficient mice; one pair of 6-week-old WT and *Mll5*$^{-/-}$ male littermates. **(f)** Survival curves of lethally irradiated recipients transpanted with WT and *Mll5*$^{-/-}$ BM (n=10). Data were analyzed by log-rank (Mantel-Cox) test. (**** $p<0.0001$).

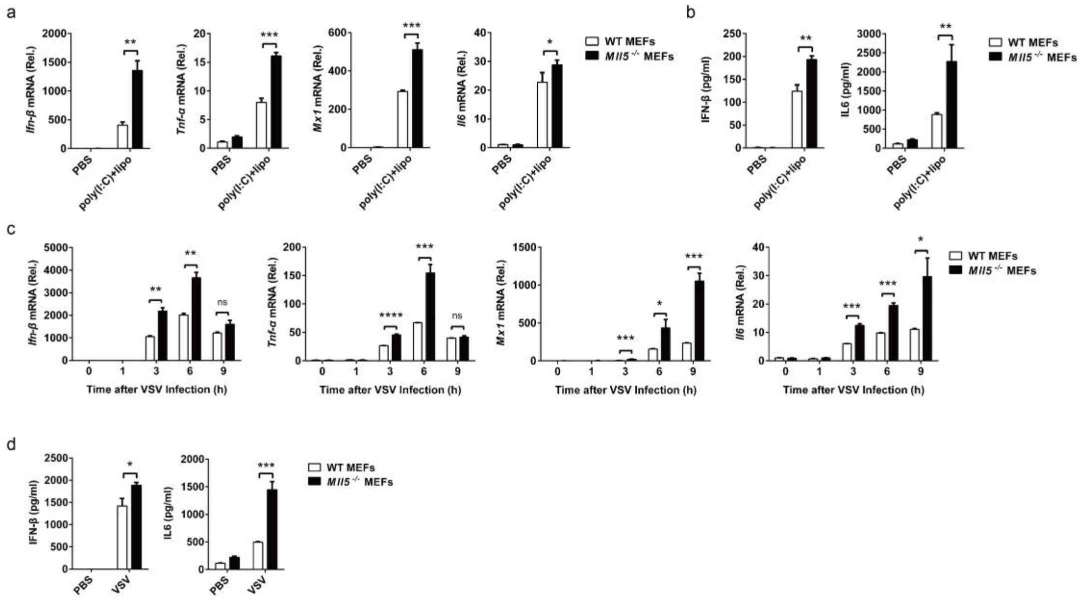

Supplementary Figure 2. MLL5 selectively suppresses RLR-mediated antiviral innate immune response in mouse embroynoic fibroblasts. **(a)** Expression of *Ifn-β*， *Tnf-α, Mx1* and *Il6* mRNA in MEFs from WT or *Mll5*$^{-/-}$ mice stimulated with intracellular poly(I:C) (1 μg/ml) for 6 h. *Gapdh* served as a control. **(b)** ELISA quantification of IFN-β and IL-6 secretion in MEFs treated as in **a**. **(c)** Expression of *Ifn-β*， *Tnf-α, Mx1* and *Il6* mRNA in MEFs from WT or *Mll5*$^{-/-}$ mice infected with VSV-GFP (MOI:1) . *Gapdh* served as a control. **(d)** ELISA quantification of IFN-β and IL-6 secretion in MEFs from WT or *Mll5*$^{-/-}$ mice infected with VSV-GFP (MOI:1) for 6 h. Data were from three independent experiments and were analyzed with the Student's *t*-test (two-tailed) and were presented as mean ± SD. (* $p<0.05$， ** $p<0.01$， *** $p<0.001$, **** $p<0.0001$).

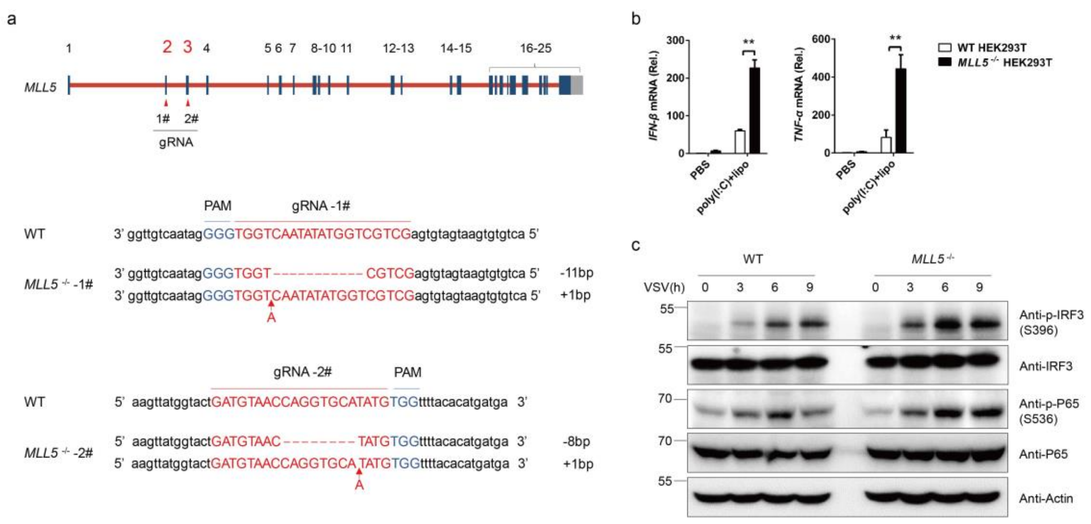

Supplementary Figure 3. MLL5 suppresses RLR-mediated immune response in HEK293T cells. **(a)** Graphic representation of strategy used to generate *MLL5*$^{-/-}$ HEK293T cells (upper) and DNA sequence of *MLL5*$^{-/-}$ HEK293T cells (bottom). gRNA (red) and PAM (blue) sequences locus in exon 2 and 3 of *MLL5* gene. The column on the right indicates the number of inserted or deleted bases. **(b)** Expression of *IFN-β* and *TNF-α* mRNA in WT and *MLL5*$^{-/-}$ (2#) HEK293T cells stimulated with intracellular poly(I:C) (1 μg/ml) for 6 h. *GAPDH* served as a control. **(c)** Immunoblot analysis of p-IRF3 and P65 in WT and *MLL5*$^{-/-}$ (2#) HEK293T cells upon infection with VSV-GFP (MOI:1) for indicated times. Actin served as a loading control. Data were representative of three independent experiments with similar results **(c)** or were from three independent experiments **(b)** and were analyzed with the Student's *t*-test (two-tailed) and were presented as mean ± SD. (** p<0.01).

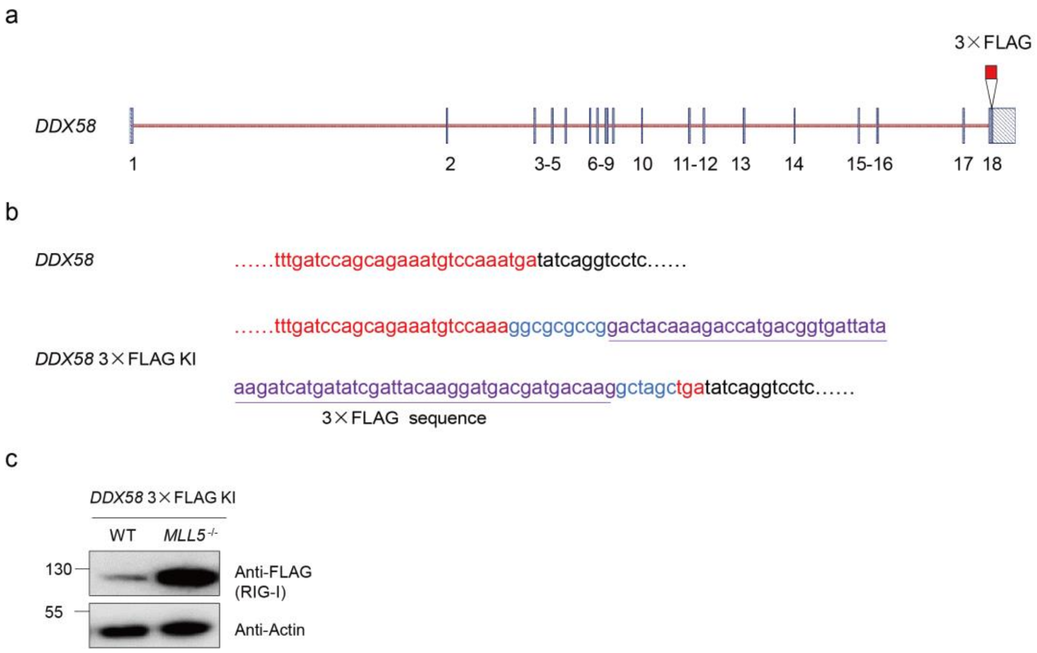

Supplementary Figure 4. Generation of FLAG-tagged endogenous RIG-I protein in HEK293T cells. **(a)** Schematic of 3×FLAG coding sequence insertion in *DDX58* gene in WT and $MLL5^{-/-}$ HEK293T cells. **(b)** DNA sequence around the stop codon (tga, red letters) of *DDX58* gene in WT cells (upper) and the 3×FLAG knock-in cells (bottom). Purple indicates the 66-bps 3×FLAG coding sequence. Red indicates the coding sequence of *DDX58*. Blue indicates the restriction enzyme cutting sites. **(c)** Immunoblot analysis of FLAG-tagged RIG-I in the *DDX58* 3×FLAG knock-in WT and $MLL5^{-/-}$ HEK293T cells. Actin served as a loading control. Data are representative of three independent experiments with similar results.

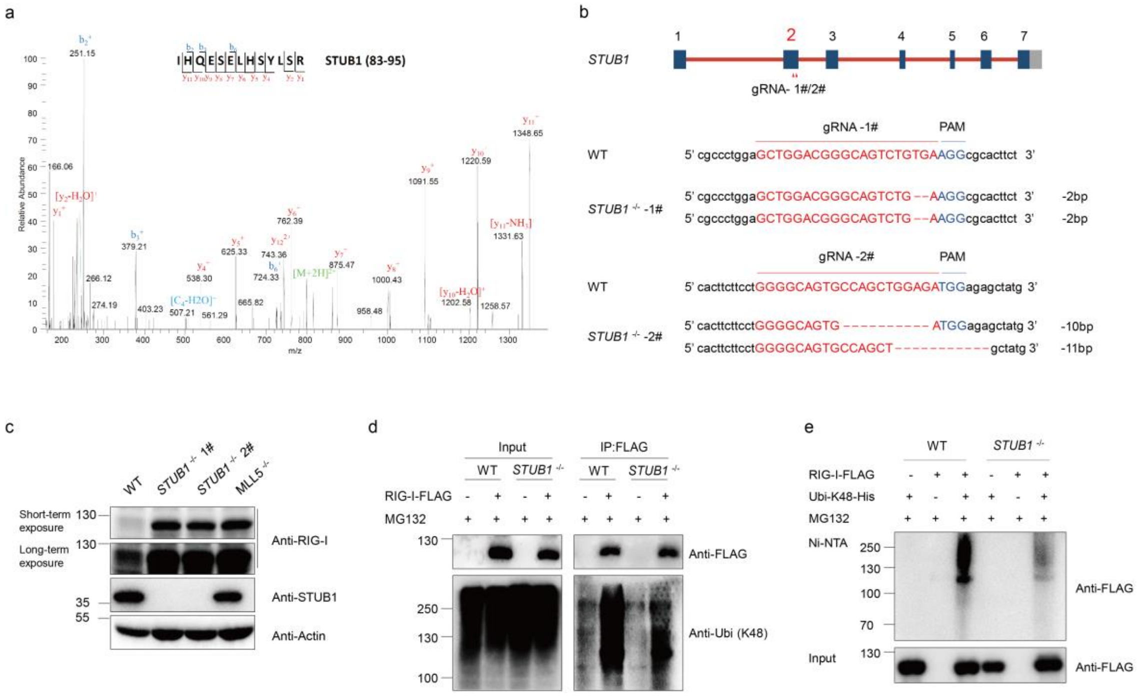

Supplementary Figure 5. Identification of STUB1 as the E3 ligase of RIG-I. **(a)** STUB1 in RIG-I-interacting proteins identified by mass spectrometry. **(b)** Graphic representation of strategy used to generate *STUB1*$^{-/-}$ HEK293T cells (upper) and DNA sequence of *STUB1*$^{-/-}$ HEK293T cells (bottom). gRNA (red) and PAM (blue) sequences locus in exon 2 of *STUB1* gene. The column on the right indicates the number of inserted or deleted bases. **(c)** Immunoblot analysis of RIG-I and STUB1 in the WT and *STUB1*$^{-/-}$ HEK293T cells. Actin served as a loading control. **(d)** Co-immunoprecipitation and immunoblot analysis of K48-linked polyubiquitination of RIG-I in WT and *STUB1*$^{-/-}$ (1#) HEK293T cells transfected with RIG-I-FLAG and treated with MG132 (5 μM) for 12 h before. **(e)** His-pull down and immunoblot analysis of K48-linked polyubiquitination of RIG-I in WT and *STUB1*$^{-/-}$ (1#) HEK293T cells cotransfected with RIG-I-FLAG and mutant ubiquitin K48-ubi-His and treated with MG132 (5 μM) for 12 h. Data are representative of three independent experiments with similar results.

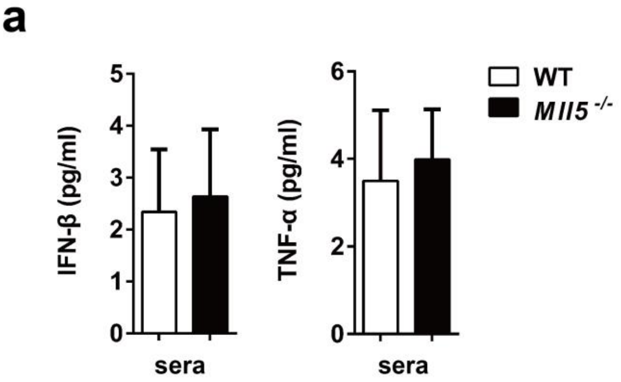

Supplementary Figure 6. ELISA quantification of IFN-β and TNF-α in sera in 6-9 weeks old WT or *Mll5*$^{-/-}$ littermates (n=3).

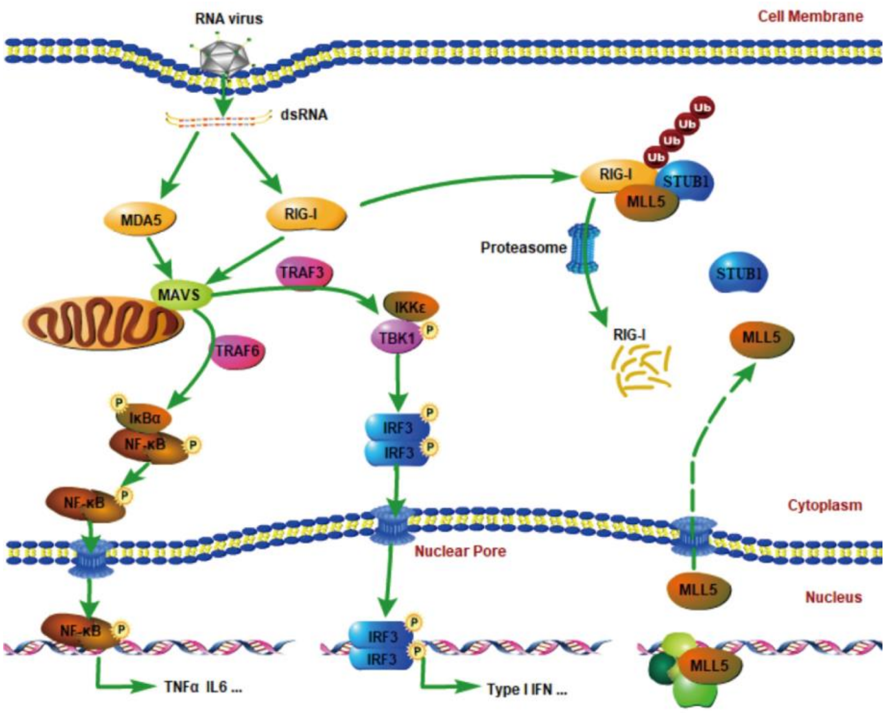

Supplementary Figure 7. Working model. A small fraction of MLL5 proteins located in the cytoplasm facilitated interaction between RIG-I and its E3 ligase STUB1 and promoted K48-linked polyubiquitination and proteasome degradation of RIG-I. MLL5 translocate from the nucleus to the cytoplasm in responses to RNA virus infection, thereby promoting degradation of RIG-I through E3 ubiquitin ligase STUB1.

a

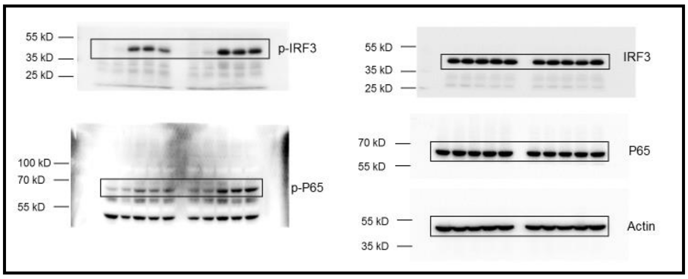
55 kD
35 kD
25 kD
p-IRF3
IRF3
100 kD
70 kD
55 kD
p-P65
P65
Actin

b

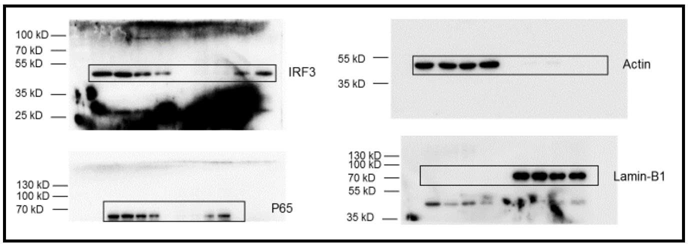
100 kD
70 kD
55 kD
35 kD
25 kD
IRF3
Actin
130 kD
P65
Lamin-B1

c

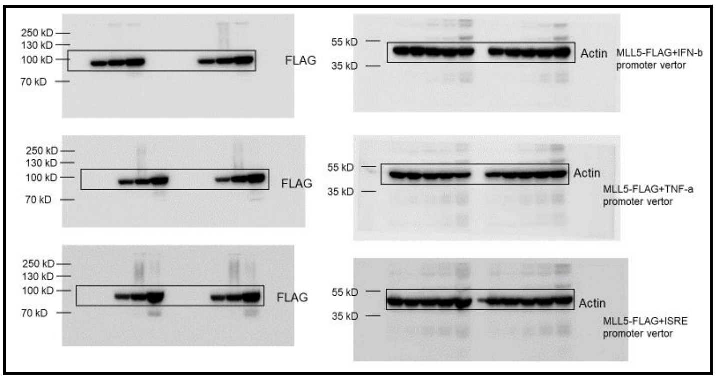
250 kD
130 kD
100 kD
70 kD
FLAG
55 kD
35 kD
Actin
MLL5-FLAG+IFN-b promoter vertor
MLL5-FLAG+TNF-a promoter vertor
MLL5-FLAG+ISRE promoter vertor

d

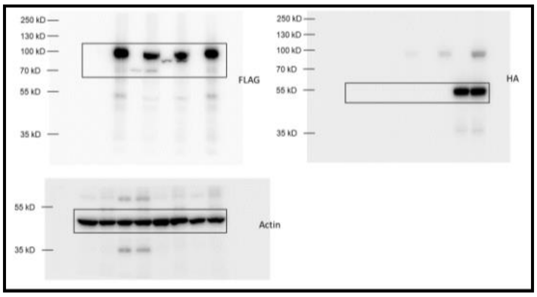
FLAG
HA
Actin

e

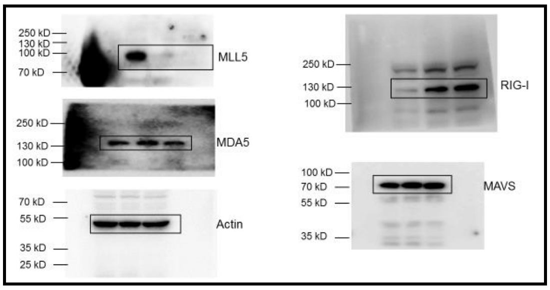
MLL5
MDA5
Actin
RIG-I
MAVS

f

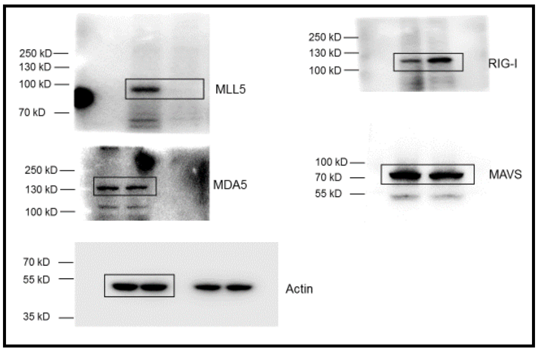
MLL5
MDA5
Actin
RIG-I
MAVS

g

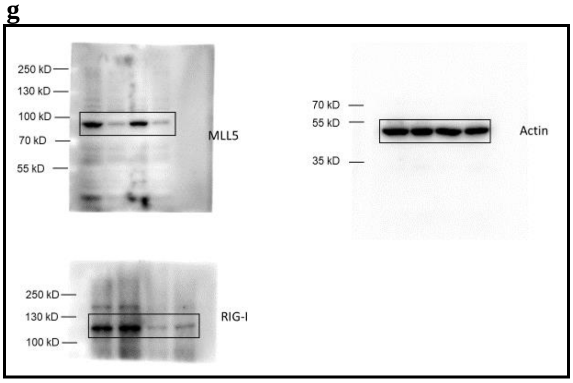
MLL5
Actin
RIG-I

h

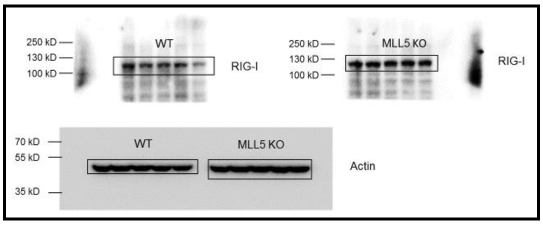
250 kD
130 kD
100 kD
WT
RIG-I
MLL5 KO
RIG-I
70 kD
55 kD
35 kD
WT
MLL5 KO
Actin

i

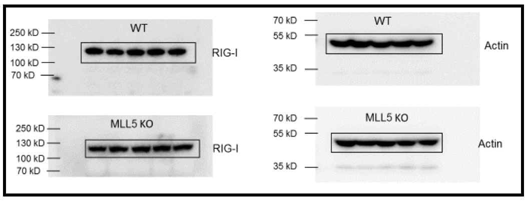
250 kD
130 kD
100 kD
70 kD
WT
RIG-I
MLL5 KO
RIG-I
70 kD
55 kD
35 kD
WT
Actin
MLL5 KO
Actin

j

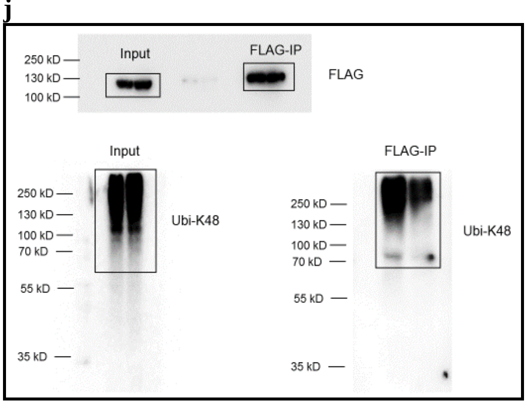
250 kD
130 kD
100 kD
Input
FLAG-IP
FLAG
Input
FLAG-IP
250 kD
130 kD
100 kD
70 kD
55 kD
35 kD
Ubi-K48
Ubi-K48

k

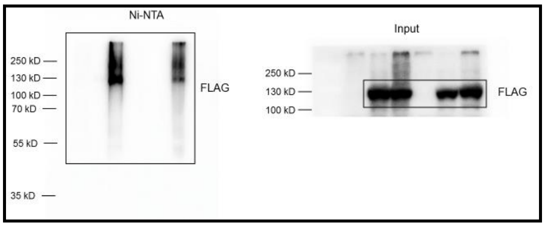
Ni-NTA
250 kD
130 kD
100 kD
70 kD
55 kD
35 kD
FLAG
Input
250 kD
130 kD
100 kD
FLAG

l

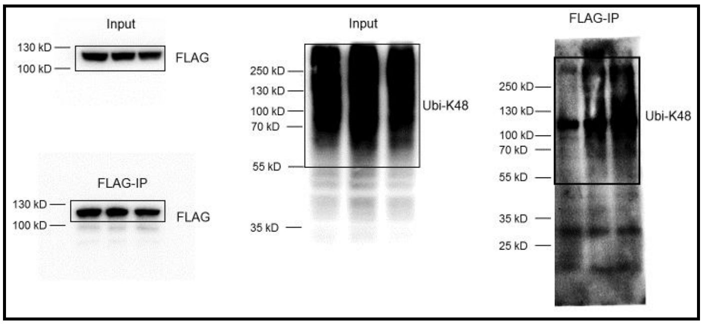
Input
130 kD
100 kD
FLAG
FLAG-IP
FLAG
Input
250 kD
130 kD
100 kD
70 kD
55 kD
35 kD
Ubi-K48
FLAG-IP
250 kD
130 kD
100 kD
70 kD
55 kD
35 kD
25 kD
Ubi-K48

m

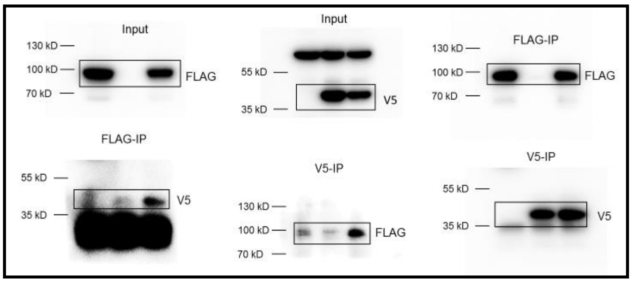
Input
130 kD
100 kD
70 kD
FLAG
Input
55 kD
35 kD
V5
FLAG-IP
FLAG
FLAG-IP
55 kD
35 kD
V5
V5-IP
130 kD
100 kD
70 kD
FLAG
V5-IP
55 kD
35 kD
V5

n

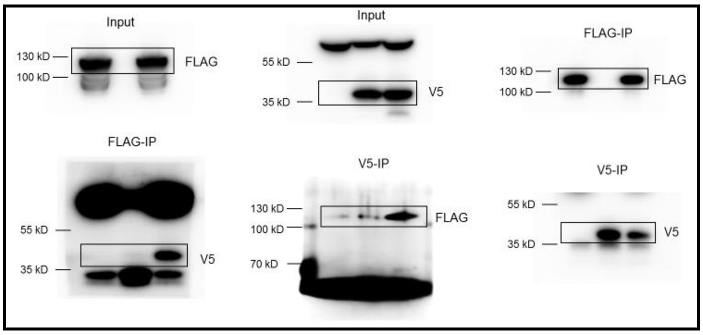
Input
130 kD
100 kD
FLAG
Input
55 kD
35 kD
V5
FLAG-IP
130 kD
100 kD
FLAG
FLAG-IP
55 kD
35 kD
V5
V5-IP
130 kD
100 kD
70 kD
FLAG
V5-IP
55 kD
35 kD
V5

o

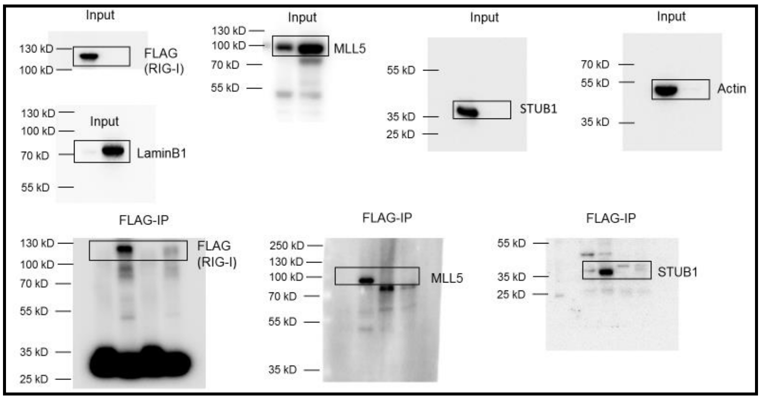
Input
130 kD
100 kD
FLAG (RIG-I)
MLL5
70 kD
55 kD
STUB1
35 kD
25 kD
Actin
LaminB1
FLAG-IP
250 kD

p

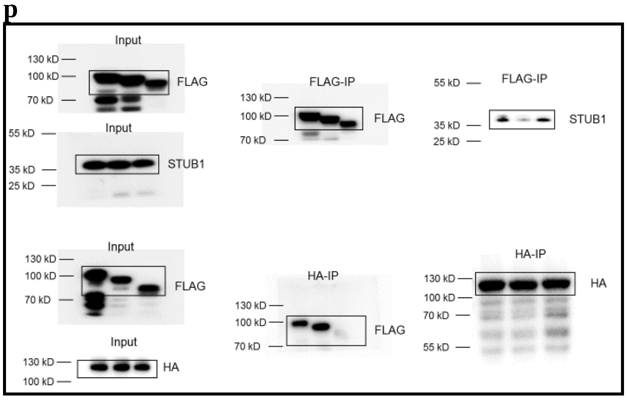
Input
FLAG
FLAG-IP
STUB1
HA-IP
HA
130 kD
100 kD
70 kD
55 kD
35 kD
25 kD

q

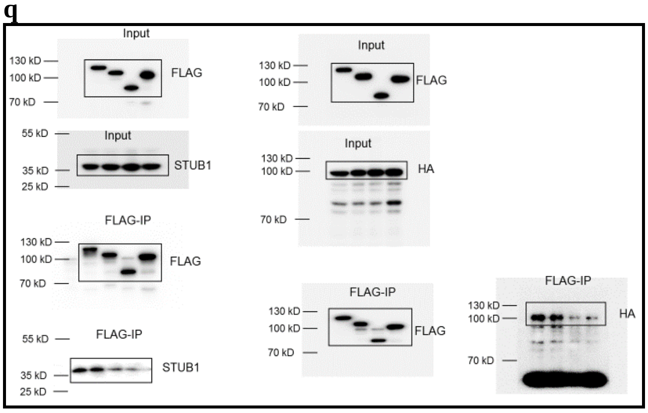
Input
FLAG
STUB1
HA
FLAG-IP
130 kD
100 kD
70 kD
55 kD
35 kD
25 kD

r

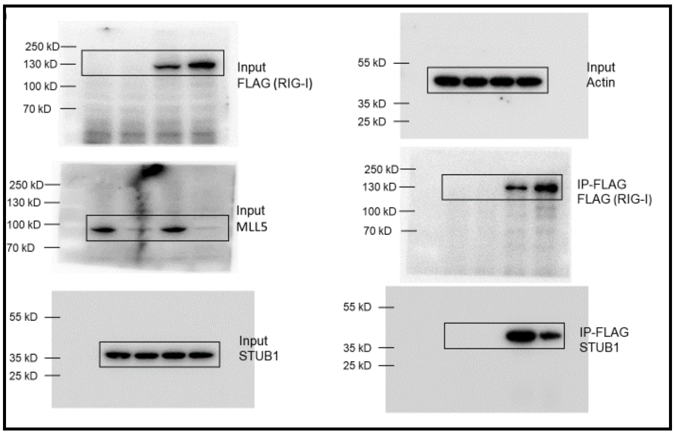
Input
FLAG (RIG-I)
Actin
MLL5
STUB1
IP-FLAG
250 kD
130 kD
100 kD
70 kD
55 kD
35 kD
25 kD

s

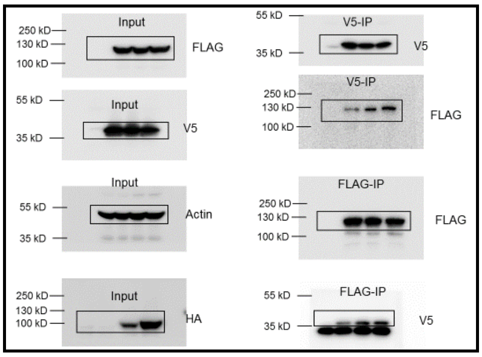

t

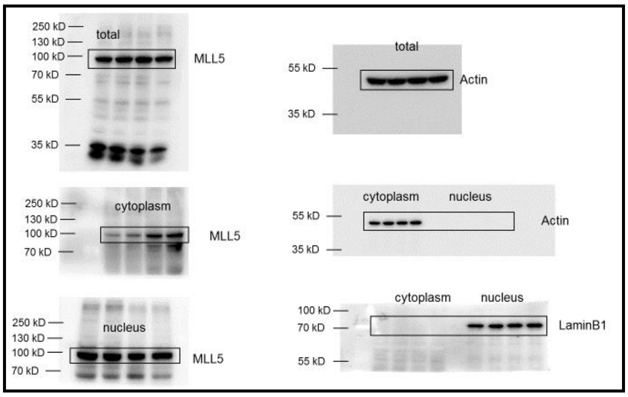

u

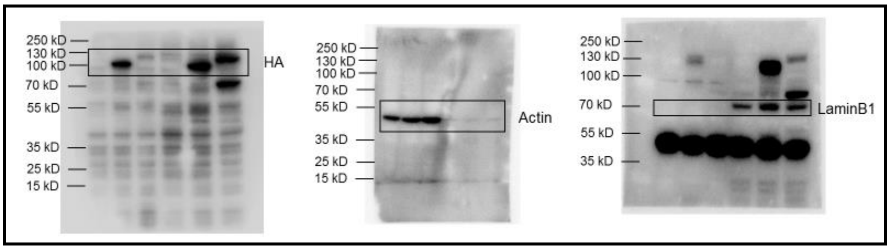

v

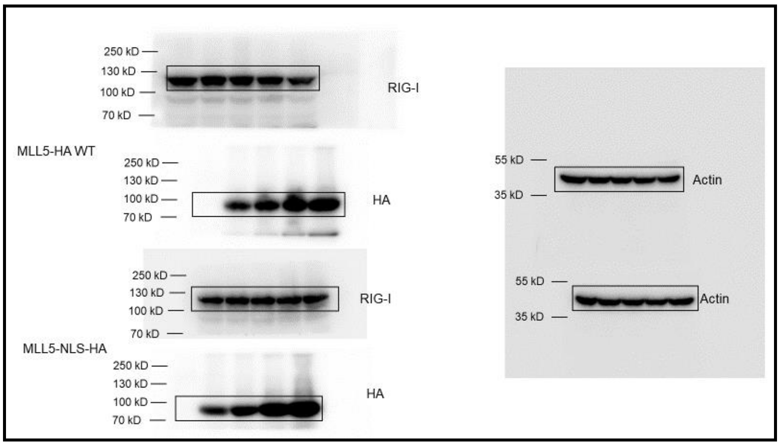

w

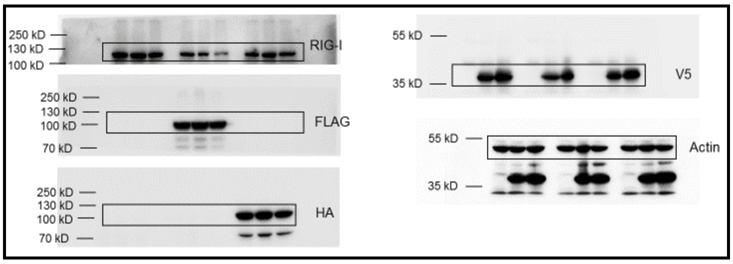

Supplementary Figure 8. Uncropped dcans of the western blots related to main figures. **(a-c)** related to Figure 2 a-c, **(d-g)** related to Figure 4 a-d, **(h-l)** related to Figure 4 g-k, **(m-o)** related to Figure 5 a-c, **(p-q)** related to Figure 5 e and f, **(r-s)** related to Figure 6 a and b, **(t-w)** related to Figure 7 a, c, e and f.

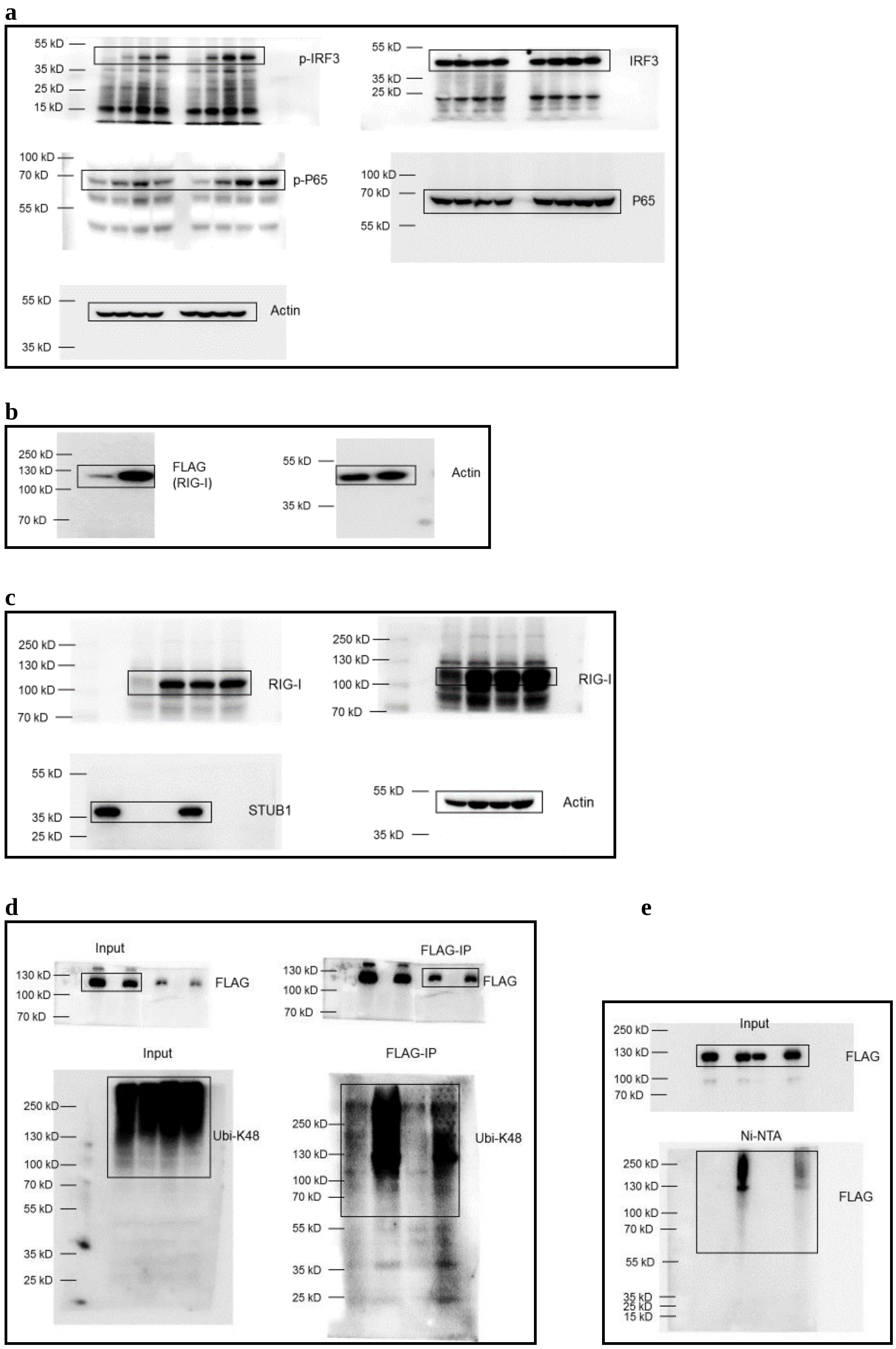

Supplementary Figure 9. Uncropped dcans of the western blots related to Supplementary Figures. **(a)** related to Supplementary Figure 3 c, **(b)** related to Supplementary Figure 4 c, **(c-e)** related to Supplementary Figure 5 d-f.

Supplementary Table 1. Relative band densities indicating RIG-I protein levels in WT and *MLL5*$^{-/-}$ HEK293T cells treated with CHX (100 μg/ml).

Time	0h	15h	20h	25h	30h
WT	1.0	0.90	0.68	0.69	0.60
MLL5$^{-/-}$	1.0	0.98	1.02	1.05	1.0
Time	0h	6h	12h	18h	24h
WT	1.0	0.95	0.68	0.48	0.47
MLL5$^{-/-}$	1.0	1.02	0.94	0.86	0.88

Actin served as a loading control. Band density indicating protein amount was quantified using Image J software.

Supplementary Table 2. Candidates RIG-I-interacting proteins identified by mass spectrometry.

seq count	gene name
703	DDX58
91	HUWE1
34	MLL5
41	TRIM28
40	HECTD1
29	HERC5
25	RNF135
20	TRIM21
13	TRIM25
9	UBR5
7	ECM29
6	MUL1
4	HUWE1
3	RNF138
4	HERC1
4	STUB1
3	MARCH7
3	XIAP
3	UBL4A
3	UFL1
2	KCMF1
2	DTX3L
2	RNF187
2	TRIM26
2	RANBP2
2	CBX4

Supplementary Table 3. Relative band densities indicating MLL5 protein levels in the nuclear and cytoplasmic fractions in WT HEK293T cells infected with VSV-GFP (MOI:1).

Time (VSV-GFP)	0h	5h	10h	15h
Cytoplasm	1.0	1.19	1.65	1.70
Nucleus	1.0	0.96	0.83	0.85
Time (VSV-GFP)	0h	4h	8h	
Cytoplasm	1.0	1.10	1.62	
Nucleus	1.0	0.95	0.87	

Actin served as a cytoplasmic control. Lamin B1 served as a nuclear protein control. Band density indicating protein amount was quantified using Image J software.

Supplementary Table 4. Relative band densities indicating RIG-I protein levels in *MLL5*$^{-/-}$ HEK293T cells transfected with different amount of MLL5-HA or MLL5-NLS-HA plasmids (0, 300, 600, 1000 and 2000 ng).

Amount of Plasmids	0	300ng	600ng	1000ng	2000ng
MLL5-HA	1.0	0.46	0.46	0.49	0.37
	1.0	0.85	0.80	0.76	0.75
MLL5-HA-NLS	1.0	1.22	1.16	1.02	0.93
	1.0	1.04	1.08	1.02	1.0

Actin served as a loading control. Band density indicating protein amount was quantified using Image J software.
